# Supplementary material for: Prospective, Multicenter Phase II Trial of Non-Pegylated Liposomal Doxorubicin Combined with Ifosfamide in First-Line Treatment of Advanced/Metastatic Soft Tissue Sarcomas
Source: Cancers (Basel). 2023 Oct 18;15(20):5036. doi: 10.3390/cancers15205036 (PMC10605752; doi:10.3390/cancers15205036)
Supplement: Supplementary file 1 [file cancers-15-05036-s001.zip › cancers-2639170-supplementary.pdf]

Table S1: Grade 3-4 adverse events (43 patients).

Level	Neutropenia	Febrile Neutropenia	Thrombocytopenia	Anaemia	Asthenia	Nausea-Emesis	Constipation	Liver	kidney
0	2	N	0	1	1	1	0	2	2
0	3	N	1	2	1	1	0	1	0
0	4	N	0	1	1	1	1	0	0
0	0	N	1	2	1	2	1	0	0
0	0	N	0	1	1	1	1	0	0
0	4	N	2	2	2	2	1	0	0
0	0	N	0	1	1	1	1	0	0
0	2	N	0	1	1	1	1	0	0
0	2	N	2	2	2	2	0	0	0
0	4	Y	0	1	1	2	1	0	0
0	4	N	2	2	1	2	1	2	0
0	4	N	0	2	2	1	0	0	0
0	4	N	0	2	3	0	2	0	0
0	4	N	1	1	3	1	0	0	0
0	0	N	0	0	1	1	2	0	0
0	0	N	0	0	2	2	1	0	0
0	3	N	0	2	1	1	1	0	0
0	4	N	3	1	2	1	0	0	0
0	3	N	0	1	1	0	1	0	0
0	4	N	4	3	3	2	2	0	0
0	4	N	0	0	1	0	0	0	0
0	4	Y	4	2	1	2	1	0	0
0	3	N	1	0	1	2	1	0	0
0	2	N	0	2	1	1	1	0	0
0	4	N	0	2	1	2	0	0	0
0	0	N	0	0	0	1	0	0	0
0	1	N	0	2	1	1	1	0	0
0	4	N	2	2	2	1	1	0	0
0	3	N	2	1	1	0	1	1	0
0	4	N	4	2	2	1	1	0	0
0	4	Y	4	3	2	0	0	0	0
0	0	N	0	1	1	1	0	0	0
0	4	N	2	2	3	1	0	0	0
0	4	Y	1	2	1	1	0	0	0
0	0	N	1	0	0	1	0	0	0
0	4	N	2	2	0	1	0	0	0
0	2	N	2	3	0	1	0	3	0
0	2	N	0	2	2	2	1	1	0
0	3	N	2	1	0	2	0	0	0
0	4	N	1	3	2	1	0	0	0
0	4	Y	1	2	3	1	0	1	0
0	4	N	0	1	2	1	0	1	0
0	0	N	0	3	0	0	0	0	0

The table displays data on Grade 3-4 toxicities in 43 patients who do not require dose reduction. Level 0: the patients do not receive dose reduction; Y = toxicity present; N= toxicity not present; Two patients who received only one cycle of therapy were not included in this analysis.

Table S2: Grade 3-4 adverse events (35 patients).

Level	Neutropenia	Febrile Neutropenia	Thrombocytopenia	Anaemia	Asthenia	Nausea-Emesis	Constipation	Liver	kidney
1	4	Y	4	3	2	1	2	0	1
1	4	N	0	3	0	1	2	0	0
1	4	N	4	3	0	1	1	0	0
1	4	N	0	3	1	1	0	0	0
1	3	N	1	2	2	1	0	0	0
1	4	N	0	3	2	2	0	0	0
1	4	N	1	3	1	1	1	0	0
1	4	Y	3	3	2	1	1	0	0
1	4	Y	1	2	3	3	2	0	0
1	4	N	4	3	2	0	1	0	0
1	4	N	2	3	3	2	2	0	2
1	3	N	0	1	1	0	0	0	0
1	4	Y	3	3	3	3	1	0	3
1	3	N	4	2	1	1	0	0	0
1	4	N	3	2	2	2	2	0	2
1	4	Y	4	2	1	2	2	0	0
1	4	N	3	2	2	1	0	0	0
1	4	Y	3	2	2	1	1	0	0
1	3	N	4	3	2	1	1	0	0
1	1	N	2	2	2	3	1	0	0
1	4	N	4	3	2	0	1	0	0
1	4	N	4	3	2	1	3	0	0
1	0	N	0	3	3	2	0	0	0
1	1	N	1	1	3	1	1	0	0
1	4	N	0	2	2	2	1	0	1
1	4	Y	2	2	2	0	1	1	0
1	1	N	0	2	2	2	0	1	0
1	4	Y	3	3	3	2	2	3	0
1	4	Y	3	3	0	0	0	0	3
2	4	Y	4	3	2	2	0	0	0
2	4	Y	4	3	2	1	0	0	0
2	4	N	3	3	2	1	2	0	1
2	4	N	2	2	2	2	1	0	2
2	4	Y	1	2	0	0	0	0	0
2	4	Y	1	0	2	2	0	0	0

The table displays data on Grade 3-4 toxicities that required a dose reduction.

Level 1 corresponds to a 75% dose reduction while Level 2 corresponds to a dose reduction. Y = toxicity present N= toxicity not present
